# Misplacement of the tunnel hemodialysis catheter through the left jugular vein to the azygos vein: A case report

**DOI:** 10.1097/MD.0000000000019805

**Published:** 2020-04-10

**Authors:** Dan Cao, Zhaonan Shen, Qiaoqiao Gui, Yun He

**Affiliations:** Department of Nephrology, Chongqing Renji Hospital University of Chinese Academy of Sciences, Chongqing, China.

**Keywords:** azygos vein, hemodialysis, misplacement

## Abstract

**Rationale:**

The percutaneous catheterization of central veins is widely applied in patients with end-stage renal diseases as a permanent vascular access. To our knowledge, inadvertent placement of a hemodialysis catheter into the azygos vein through the left internal jugular vein is not described.

**Patient concerns:**

A 72-year-old female patient was admitted to the hospital for replacement of another new tunneled hemodialysis catheter due to poor flow in the left internal jugular vein tunneled catheter during hemodialysis.

**Diagnosis:**

The catheter tip was incorrectly positioned into the azygos vein as confirmed by conventional anteroposterior and lateral chest radiographs.

**Interventions:**

The catheter was removed and replaced under Digital Subtraction Angiography.

**Outcomes:**

The catheter tip was finally placed in the proper position.

**Lessions:**

The insertion of central vein catheterization is not always in suitable position especially through left jugular vein in hemodialysis patients. DSA technology should be performed to confirm the correct position of the tip and to ensure good blood flow.

## 1 Introduction

It is widely believed that primary arteriovenous fistula is the optimal vascular access for hemodialysis. However, percutaneous tunnel catheters have been selected for aging dialysis populations who have difficulty in fistula formation or short life expectancy.^[1]^ Jugular vein catheterization is considered to have the lowest risk of complications. In order to obtain a good dialysis effect, the tip of the catheter should be placed in the superior vena cava, and the cavoatrial junction.^[2]^ The venous placement of the hemodialysis catheter in the azygos vein is rare.^3^,^4^,^5^ And in the reported three cases, the hemodialysis catheter was all incorrectly positioned in the right jugular vein. Here, in this study, we present a case of an unusual cannulation of the azygos vein with a hemodialysis catheter through the left jugular vein.

## 2 Case report

A 72-year-old female patient was admitted to the hospital for replacement of another new tunneled hemodialysis catheter due to a lack of flow in the left internal jugular vein tunneled catheter during hemodialysis. Prior to this, she had endured quite a few catheter thrombolysis, but the results were not satisfactory. The underlying disease that caused her uremia was diabetic nephropathy. Her vascular condition did not meet the original requirements for establishing arteriovenous fistula. And, a year ago, her right jugular vein tunnel was pulled out due to a catheter infection. Since then, she had used the left jugular vein catheter as a vascular access for the dialysis.

**Figure 1:**
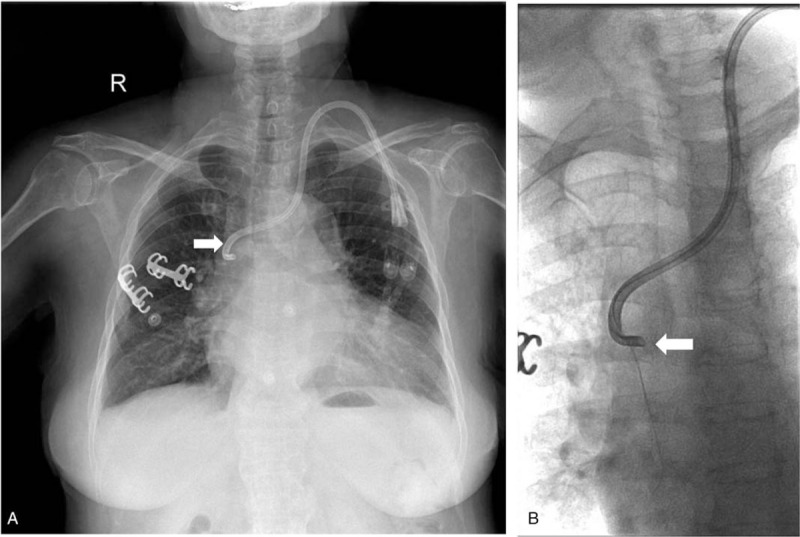
Analysis of catheter tip malposition. (A) Chest radiograph showing catheter tip malposition close to the right tracheobronchial angle structure (azygos vein) like a fishhook (arrow). (B) By injecting contrast into the venous port, DSA confirms that the tip of the catheter is placed in the azygos vein (arrow). DSA = digital subtraction angiography.

**Figure 2:**
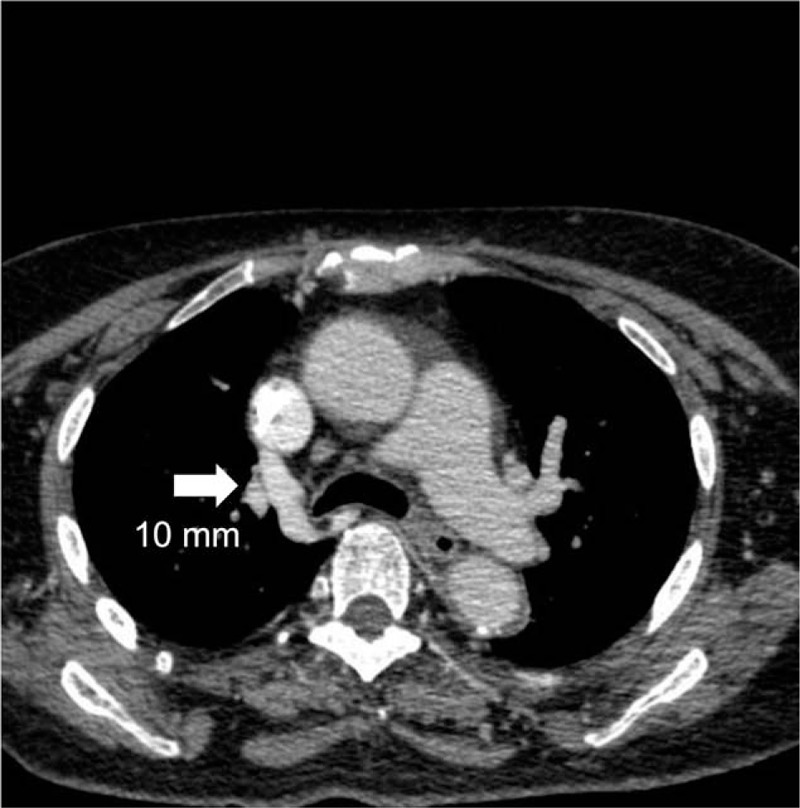
CT scan of azygous vein. The azygos vein was thickened with ostial size of 10 mm (arrow).

**Figure 3:**
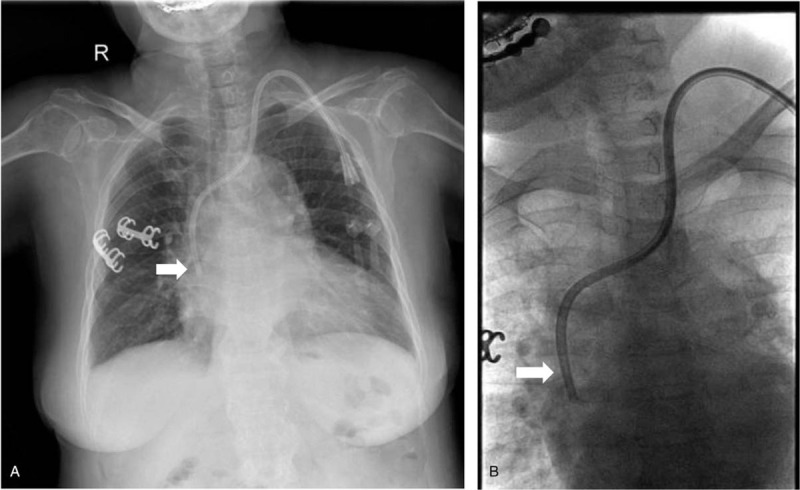
Analysis of catheter tip re-position. (A) Chest radiograph showing catheter tip has reached the correct position in the cavoatrial junction after being modulated under DSA (arrow). (B) DSA shows the hemodialysis catheter after redeployed within the right atrium (arrow). DSA = digital subtraction angiography.

By placing the guidewire into the catheter's venous port as a guide and establishing a new percutaneous tunnel, we successfully placed a new tunnel catheter (14.5 Fr, asymmetric tip, 36 cm) and then got non-pulsatile blood flow. Conventional anteroposterior and lateral chest radiographs were performed to assess catheter tip position following the catheter placement. However, we found that the catheter tip was incorrectly positioned into the azygos vein (Fig. 1A). It was noted that the catheter tip kinked obviously, and inclined medially at the right tracheobronchial angle. This was further confirmed by injection of a contrast agent through the venous orifice of the catheter (Fig. 1B). CT scan showed the thickening azygos vein, the ostial size of which had reached 10 mm (Fig. 2). After removing the catheter and replacing it under the DSA (Digital Subtraction Angiography), we finally placed the catheter tip in the proper position (Fig. 3). Since the replacement, subsequent dialysis sessions have been successful, with no further flow-related issues.

## 3 Discussion

The right jugular vein catheterization is regarded as the desirable access for hemodialysis because of its anatomic structure. Inadvertent placement of a hemodialysis catheter into the azygos vein is not desired for dialysis. The azygos vein is occasionally chosen for the alternative pass way only under conditions of serious venous occlusion.^[6]^ However, it is less preferred due to its comparatively small vessel caliber and blood flow direction.^[7]^ Risk factors for malpositioning into the azygos vein include left internal jugular venous access, obesity, deficient in ultrasonic guidance, congenital variants or subtotal stenosis of other central veins, which lead to collateral circulation expansion.^[5]^ Juan et al reported a misplacement of a dialysis catheter in the azygos vein through the right jugular access, which was caused by the high right atrial pressure as a result of fluid overload and a subsequent enlargement of the ostium of the azygos vein.^[3]^ Sometimes, back pain also indicates the presence of catheter tip malpositioning into the azygos vein.^[8]^ Without chest radiograph, it is difficult to find the malposition into the azygos vein. In our case, the right jugular venous stenosis due to the placement of the original catheter can account for the growth of the azygos vein.

Azygos vein is 6 to 8 mm in normal physiological circumstances.^[9]^ But in our case, its ostial size has reached 10 mm. This has increased the chance of inadvertent cannulation during hemodialysis catheter insertion. Meanwhile, the longer course and more transverse disposition of the left brachiocephalic increase the possibility of malpositioning. Ewa et al demonstrated that with the blind insertion of tunneled hemodialysis catheters, the risk of catheter tip malposition was significantly higher with the left side insertion.^[1]^ Therefore, in our clinical practice, chest radiograph is always carried out after central vein catheters placement. We found a sharp connection like a fishhook near the right tracheobronchial angle, which is a constant anatomical landmark^[5]^ and where the azygos vein opens into the superior vena cava. This phenomenon indicated that the tip of the catheter was mispositioned into the azygos vein. In some cases, venous blood can also be easily aspirated from the azygos veins, which can lead to a clinical impression of proper catheter tip placement. But this could be easily detected by the radiographic technology. After replacing the tip position of the tunneled hemodialysis catheter under DSA (digital subtraction angiogram), we observed that the tip reached the right atrium opening.

In summary, central venous catheterization may not reach the desirable position all the time. As reported in our case, the tip of the catheter may be mispositioned, especially from the left jugular vein. We should use the chest radiograph to confirm the correct position of the tip according to the anatomic landmark. DSA technology should be used to modulate its position and solve the dilemma.

## Acknowledgments

The authors thank all the members in Nephroly department and Radiology intervention department.

## Author contributions

Dan Cao collected and interpreted the data and prepared the manuscript. Zhaonan Shen and Qiaoqiao Gui collected the data and searched the literature. Yun He designed the study and edited the manuscript. All the authors have read and approved the manuscript and believe that the manuscript represents honest work.

**Conceptualization:** Yun He.

**Data curation:** Dan Cao.

**Formal analysis:** Dan Cao.

**Methodology:** Dan Cao, Zhaonan Shen, Qiaoqiao Gui.

**Software:** Dan Cao, Zhaonan Shen, Qiaoqiao Gui.

**Writing – original draft:** Dan Cao.

**Writing – review & editing:** Yun He.

Yun He orcid: 0000-0002-6611-0400.
